# A New Animal Model for Pathological Subcutaneous Fibrosis: Surgical Technique and *in vitro* Analysis

**DOI:** 10.3389/fcell.2020.00542

**Published:** 2020-07-30

**Authors:** Andrea Marchesini, Francesco De Francesco, Monica Mattioli-Belmonte, Nicola Zingaretti, Valentina Riccio, Fiorenza Orlando, Barbara Zavan, Michele Riccio

**Affiliations:** ^1^Department of Reconstructive Surgery and Hand Surgery, AOU “Ospedali Riuniti”, Ancona, Italy; ^2^Department of Clinical and Molecular Sciences, Università Politecnica delle Marche, Ancona, Italy; ^3^Clinic of Plastic and Reconstructive Surgery, Department of Medical Area (DAME), Academic Hospital of Udine, University of Udine, Udine, Italy; ^4^Veterinary Medical School, University of Camerino, Camerino, Italy; ^5^Experimental Animal Models for Aging Unit, Scientific Technological Area, IRCCS INRNCA, Ancona, Italy; ^6^Department of Medical Sciences, University of Ferrara, Ferrara, Italy

**Keywords:** pathological fibrosis, wound model, animal model, scar, talc

## Abstract

Fibrosis is a condition that affects the connective tissue in an organ or tissue in the restorative or responsive phase as a result of injury. The consequences of excessive fibrotic tissue growth may lead to various physiological complications of deformity and impairment due to hypertrophic scars, keloids, and tendon adhesion without understating the psychological impact on the patient. However, no method accurately quantifies the rate and pattern of subcutaneous induced hypertrophic fibrosis. We, therefore, devised a rodent excisional model to evaluate the extent of fibrosis with talc. Tissue specimens were set on formalin, and paraffin sections for histological, immunohistochemical, and molecular analysis talc was used to induce the fibroproliferative mechanism typical of hypertrophic scars. This pathway is relevant to the activation of inflammatory and fibrotic agents to stimulate human hypertrophic scarring. This model reproduces morpho-functional features of human hypertrophic scars to investigate scar formation and assess potential anti-scarring therapies.

## Introduction

Keloids, tendon adhesion and other diseases resulting from excessive connective tissue formation to a pathological degree are challenging undertakings for plastic and orthopedic surgeons who are not able to guarantee satisfactory outcomes.

Fibrosis is a condition that occurs in connective tissue and tissue as a typical physiological consequence of the reparative mechanism that follows injury. Hypertrophic fibrosis increases the tissue volume due to an anomalous propagation of fibroblast cells and aggregation of extracellular elements such as collagen. The augmented volume of fibrotic tissue may lead to important physical and psychological disabilities and impairments (Brown et al., 2008; Finnerty et al., 2016; Zhang et al., 2018). The healing process of a tissue injury is characterized by three concurring mechanisms: inflammation, proliferation and remodeling (Reinke and Sorg, 2012).

These phases occur at onset of the lesion with hematoma, movement of cells from the surrounding tissue and the activation of inflammatory mediators. During the proliferative phase, new blood vessels form from pre-existing vessels followed by the deposition of extracellular compounds. During remodeling the extracellular matrix is characterized by the development of elements and affections, which consequently lead to the deposition of proteoglycan and collagen associated to scarring and rigidity in the joints (Gurtner et al., 2008).

Hypertrophic scars of the dermis are caused by the aggregation of collagen cross-links and are responsible for tissue hardening as well as an excess of collagen in the area above the surrounding skin or the area around the initial wound (Sidgwick and Bayat, 2011). Kischer and Brody (1981) defined collagen nodules as the structural unit of hypertrophic scars and keloids (Liu et al., 2012). The nodules are devoid of mature scars and present an advanced organization and precise orientation of significantly dense fibroblasts and unidirectional collagen fibrils. Moreover, a hypertrophic scar possesses a rich vasculature (in relation to scar age) which differentiates it from normal skin. The scar besides, presents an elevated mesenchymal cell density, and a deep epidermal cell layer. Keloidal scars consist of type III (early) or type I (late) collagen differing according to the maturity (Lee and Jang, 2018).

Flexor tendon reconstruction is a challenging issue if the injury presents in zone II of the hand. Common occurrences are adhesions involving the areas between the tendon and the surrounding tissue and limitations in the range of motion of the finger with subsequent contraction of the joint. Histologically, tendon fascicles contain type I collagen fibers and spiraling bundles of mature fibroblasts or tenocytes that are long and narrow. Adhesions between the tendon and the surrounding tissue area is common in injuries that mainly heal extrinsically, conversely to intrinsic cellular healing which will present with fewer adhesions and reduced density (Mass and Tuel, 1991). However, adhesion formation is also related to tendon suture, sheath damage and immobilization following surgery which are inevitable consequences of the injury itself and the reconstruction phase (Elliot et al., 2007; Riccio et al., 2010).

Clinical examples of hypertrophic fibrosis also include Ledderhose's syndrome, Peyronie's disease, or more frequently Dupuytren's disease. Histological analyses have shown a higher number of collagen III deposits compared to collagen I deposits, and an increase in collagen hydroxylation and glycosylation, myofibroblasts and myoglobin proteins. Despite our extensive knowledge regarding the biology of cytokines, growth factors and the altered expression of several genes, the etiology and pathogenesis of the disease are still unclear (Bisson et al., 2004; Chen et al., 2011) with gray areas especially in the biology and treatment of proliferative fibrotic tissue. The lack of literature is partly a result of inaccurate, inaccessible, and unreproducible animal models for the study of hypertrophic fibrosis (Huang et al., 2013).

The role of the various cell types and cytokines have been analyzed in hypertrophic scarring via *in vitro* studies investigating cell lines and primary cell culture. However, the efficacy of the procedure is seriously restricted to the differing conditions in controlled cultures and the wound-healing contexts considering, in particular, the numerous and intricate relations among types of cells, cytokines and extracellular matrix components. The search for optimal animal studies are underway but are currently being disputed (Dohi et al., 2019), due to the phylogenetic differences in wound healing (Ramos et al., 2008). Rodent and human skin differ considerably as in hair covering density—rodent hair has a shorter growth cycle compared to human hair; dermal papillae/apocrine gland variations; the differences in the panniculus carnosus (in humans it will rapidly aid in wound contraction) (Wong et al., 2011).

Attempts to develop animal models have always been a challenging and not always successful enterprise but despite the drawbacks numerous experimental animal models have indeed evolved. In particular, Morris et al. (1997) studied proliferative scar progress in a rabbit ear abrasion and transplantation of human proliferative scar tissue (Morris et al., 1997). Moreover, Aksoy et al. (2002) developed scar hypertrophy in guinea pigs by irritation caused by coal tear application (Aksoy et al., 2002). Wound healing was also investigated by Galiano et al. (2004) and Jimi et al. (2017a) observing that wound contraction was related to skin mobility in animals As for tendon adhesion, two models are mostly used: the removal of the flexor sheath with exposure of the underlying flexor digitorum profundus and superficialis system, or the association of a complete or partial tendon laceration. New Zealand White Rabbit toes are commonly adopted as well as chicken hind-paws or canine forepaws (Porat et al., 1980; de Wit et al., 2008; Taguchi et al., 2009).

Thoracoscopic talc poudrage (2.5–10 g) has been successfully used as a treatment option in reiterated pleural effusions due to the ability of talc to promote an extensive inflammatory reaction involving coagulation parameters and fibroblast proliferation (Marchi et al., 2007; Moreno-Merino et al., 2012). Therefore we hypothesized that subcutaneous talc installation could induce a chronic inflammatory reaction that promoted an irregular expansion of fibroblast cells and extracellular component aggregation resulting in the formation of a hypertrophic scar. Wound models with subcutaneous talc instillation have not been widely used, therefore we assessed an alternative procedure to correctly monitor epidermal reconstruction following subcutaneous talc instillation, to determine a standard evaluation of dermal remodeling and renewal.

Literature is currently lacking in a set assessment tool regarding subcutaneous induced hypertrophic fibrosis. The aim herein is to describe the surgical technique and to assess histologically, a new animal model for hypertrophic fibrosis.

We propose to redesign the pathological mechanism that underpin hypertrophic scarring with the use of talc.

## Materials and Methods

We conducted the study according to European and Italian Law on animal experimentation and all policies and procedures conformed to 86/609/CEE directives. Forty-eight male Wistar rats (340 ± 60 g/BW) were subjects of the study (Experimental Animal Models for Aging Units Research Department, I.N.R.C.A./I.R.R.C.S., Ancona, Italy). This animal experiment was approved by the Ethic Committee (No.1CHPL/08-13). Inbred, genetically identical rats were used. The study subjects were equally distributed into four groups. Animals were maintained in single cages and regulated for temperature and moisture level, adequate supplies of water and food were available at all times.

### Surgical Procedure and Sample Preparation

**Figure 1:**
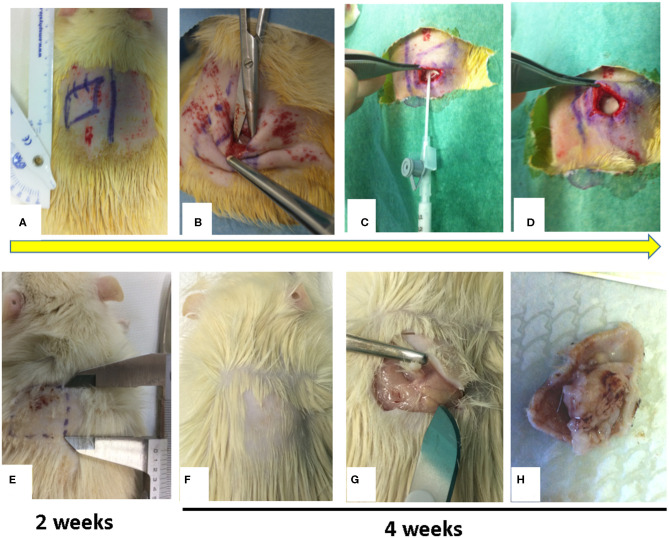
Figure showed a sample of surgical procedure. **(A)** Monoliteral cutaneous incision in the dorsal paravertebral region; **(B)** Pocket preparation between subcutaneous connective layer and the Dorsalis Magnus muscle fascia; **(C)** Injection at different concentrations of talc into the pocket; **(D)** Skin closure of pocket to avoid talc overflow; **(E)** at 2 weeks, palpatory, in the injection site there is no subcutaneous granuloma formation (the rats were not sacrificed); **(F–H)** at 4 weeks, palpatory, in the injection site, there is subcutaneous granuloma formation and after sacrificing the rat, evidence of subcutaneous granuloma formation is observed.

We administered ketamine (40 mg/ Kg) and intramuscular xylazine (5 mg/kg) to anesthetize the rats setting them face down on a warm pad. We used this combination for a constant and reliable level of immobilization and anesthesia (Fish et al., 2008). A monolateral cutaneous incision of 2 cm in length was performed, on a randomized side, in the dorsal paravertebral region just below the scapula (Figure 1A). This position prevents rats from interfering (i.e., biting or scratching) with the surgical treatment. After the cutaneous incision a 2 × 2 cm squares pocket was executed between the subcutaneous connective layer and the Dorsalis Magnus muscle fascia (Figure 1B); we also implemented scraping without cutting the underlying Dorsalis Magnus muscle fascia using a repeated passage of a surgical blade. Talc at different concentrations was injected into the cavity through a sterile syringe (Figure 1C). Skin closure was performed with close attention to preventing talc diffusion or flow out (Figure 1D).

We randomly assigned 48 rats to four groups to endeavor optimal talc concentration for a hypertrophic scar.

GROUP A: Control group, only the subcutaneous pocket and fascia scratch were performed.

GROUP B: Treatment group, 1 ml of sterile saline solution + 16 mg of SteritalcF4 was instilled into the subcutaneous pocket.

GROUP C: Treatment group, 1 ml of sterile saline solution + 160 mg of SteritalcF4 was instilled into the subcutaneous pocket.

GROUP D: Treatment group, 1 ml of sterile saline solution + 300 mg of SteritalcF4 was instilled into the subcutaneous pocket (Marchi et al., 2007).

We administered an antibiotic therapy of 75 mg/Kg of oxytetracycline daily for 6 days and Carprofen 0.4 mg/kg at 12-h intervals on the first day post-surgery. The animals were sacrificed by anesthetic surplus; in each group, 4 rats were sacrificed 2 weeks after surgery, 4 weeks after surgery and 6 weeks after surgery. The experimental sites were dissected, and full thickness samples including the overlying skin and partial underlying muscle were collected and fixed in formaldehyde 4% and embedded in paraffin.

### Gross Examination

All rats were clinically examined by a single operator at the time of 2, 4, and 6 weeks. The following parameters were assessed at each examination stage: presence of infection, wound dehiscence, size of the palpable fibrosis area and level of adherence to the palpation between skin and muscle plane. The latter parameter referring to stiffness, was assigned a value between 0 and 10, where 0 stands for no adherence; 10 stands for fixed skin to the muscle plane.

### Histological and Immunohistochemical Evaluation

For histological analyses all the specimens were sectioned (5 μm) and were stained with haematoxylin-eosin and Masson's trichrome. Antibodies were used for immunohistochemical assessment. All specimens were examined by two masked assessors via light microscopy (Nikon Eclipse 600, Milan, Italy) and NIS-Elements Microscope Imaging Software (Nikon).

To verify our hypotheses two researchers performed masked microscopic examinations to analyse the cellular response in terms of neovascularization, fibrosis and inflammation Specifically, we analyzed 3 slides per sample using light microscopy at 20× for initial magnification. Three sections of a specimen comprised each slide and we examined five fields per tissue section. A semi-quantitative investigation was used to compare Group A and B for the specific cell types: A Polymorphic Nuclear Cells (a cell with a nucleus lobed into segments and cytoplasmic granules, i.e., granulocytes); phagocytic cells (large mononuclear cells, i.e., macrophages and monocyte-derived giant cells); non-phagocytic cells (small mononuclear cells, i.e., lymphocytes, plasma cells and mast cells.); fibroblasts; endothelial cells; elastic fibers; and collagen fibers. Assessments were all conducted blindly and hence scored as follows—absence (score 0), scarce presence (score 1), moderate presence (score 2), and profuse presence (score 3). We conducted a minimum of three assessments and expressed the values accordingly—mean ± Standard Deviation.

### Real-Time PCR Array Analysis

Total RNA from biopsies was removed with the RNeasy Mini Kit (Qiagen Gmbh, Hilden, Germany), DNase digestion using the RNase-Free DNase Set (Qiagen). 800 ng of total RNA from all specimens was reverse transcribed using an RT2 First Strand kit (Qiagen Sciences, Germantown, MD USA). Real-time PCR was conducted compliant with instructions within the Rat Wound Healing RT2 Profiler PCR array (SABiosciences, Frederick, MD, USA) employing RT2 SYBR Green ROX FAST Mastermix (SABiosciences).

We performed thermal cycling and fluorescence detection via Rotor-Gene Q 100 (Qiagen), analyzing data with Excel-based PCR Array Data Analysis templates (SABiosciences). We reported the results as an expression of every target gene in the specimens collected after treatment in comparison with pre-treatment specimens in Group A and Group B.

### Statistical Analysis

Values were indicated as the mean standard error. We conducted Least square Linear regression for assessment using a computer-aided statistics program (SPSS 16.0 software, SPSS Inc., Chicago, IL, USA). A *P* < 0.05 was considered statistically significant. One-way analysis of variance (ANOVA) was used for data analyses. Repeated-measures ANOVA with a *post-hoc* analysis using Bonferroni's multiple comparison was performed. T tests were used to determine significant differences (p < 0.05). Repeatability was calculated as the standard deviation of the difference between measurements. All testing was performed in SPSS 16.0 software (SPSS Inc., Chicago, Illinois, USA) (license of the University of Padua, Italy).

## Results

### Gross Examination

No cases of infection in the surgical site nor dehiscence in the surgical wounds were observed. On clinical examination, 2 weeks after surgery, none of the four groups of rats showed subcutaneous fibrosis on palpation nor minimal adherence (Figure 1E). The rats were thus not sacrificed. At both 4 and 6 weeks after surgery in groups A and B no development of a clinically palpable fibrotic area was noted and the adherence score was always <5. On the contrary, the animals in groups C and D after 4 weeks of surgery showed a clear area of fibrosis and the development of consistent adhesions. In particular when we compared groups C and D, a gross examination revealed a stiff, fixed and thicker lesion in group D compared to group C. The size of the fibrotic area and the score of adhesions in each group were stable over time with no alteration between 4 and 6 weeks (Figures 1F–H).

Representative views of fibrotic volume and scores are displayed in Table 1. A small area was evident between the skin and the muscle tissue, which corresponded only to the injection site. Talc-injected rodents exhibited strongly induced fibrosis within 4 weeks. Notably, increasing doses of talc triggered an increasing tissue reaction. These data suggest adequacy of the one-shot injection procedure and the efficiency of talc as an inducer of murine fibrosis.

**Table 1:** Gross examination.

**Animal No**.	**Dose of talc**	**2 weeks time**				**4 weeks time**				**6 weeks time**			
		**Palpable area of fibrosis**	**Stiffness of the area**	**Infection**	**Wound dehiscence**	**Palpable area of fibrosis**	**Stiffness of the area**	**Infection**	**Wound dehiscence**	**Palpable area of fibrosis**	**Stiffness of the area**	**Infection**	**Wound dehiscence**
1	0 mg	No palpable fibrosis	2	No	No	No palpable fibrosis	3	No	No	Sacrificed at 4 weeks			
2	0 mg	No palpable fibrosis	2	No	No	No palpable fibrosis	2	No	No	No palpable fibrosis	2	No	No
3	0 mg	No palpable fibrosis	3	No	No	No palpable fibrosis	3	No	No	Sacrificed at 4 weeks			
4	0 mg	No palpable fibrosis	2	No	No	No palpable fibrosis	2	No	No	No palpable fibrosis	2	No	No
5	0 mg	No palpable fibrosis	1	No	No	No palpable fibrosis	2	No	No	Sacrificed at 4 weeks			
6	0 mg	No palpable fibrosis	3	No	No	No palpable fibrosis	3	No	No	No palpable fibrosis	3	No	No
7	0 mg	No palpable fibrosis	3	No	No	No palpable fibrosis	4	No	No	Sacrificed at 4 weeks			
8	0 mg	No palpable fibrosis	2	No	No	No palpable fibrosis	2	No	No	No palpable fibrosis	2	No	No
9	0 mg	No palpable fibrosis	3	No	No	No palpable fibrosis	3	No	No	Sacrificed at 4 weeks			
10	0 mg	No palpable fibrosis	3	No	No	No palpable fibrosis	3	No	No	No palpable fibrosis	3	No	No
11	0 mg	No palpable fibrosis	3	No	No	No palpable fibrosis	4	No	No	Sacrificed at 4 weeks			
12	0 mg	No palpable fibrosis	4	No	No	No palpable fibrosis	4	No	No	No palpable fibrosis	4	No	No
13	16 mg	No palpable fibrosis	3	No	No	No palpable fibrosis	4	No	No	Sacrificed at 4 weeks			
14	16 mg	No palpable fibrosis	2	No	No	No palpable fibrosis	2	No	No	No palpable fibrosis	2	No	No
15	16 mg	No palpable fibrosis	2	No	No	No palpable fibrosis	3	No	No	Sacrificed at 4 weeks			
16	16 mg	No palpable fibrosis	3	No	No	No palpable fibrosis	4	No	No	No palpable fibrosis	4	No	No
17	16 mg	No palpable fibrosis	3	No	No	No palpable fibrosis	3	No	No	Sacrificed at 4 weeks			
18	16 mg	No palpable fibrosis	4	No	No	No palpable fibrosis	4	No	No	No palpable fibrosis	4	No	No
19	16 mg	No palpable fibrosis	3	No	No	No palpable fibrosis	4	No	No	Sacrificed at 4 weeks			
20	16 mg	No palpable fibrosis	4	No	No	No palpable fibrosis	5	No	No	No palpable fibrosis	5	No	No
21	16 mg	No palpable fibrosis	5	No	No	No palpable fibrosis	5	No	No	Sacrificed at 4 weeks			
22	16 mg	No palpable fibrosis	3	No	No	No palpable fibrosis	4	No	No	No palpable fibrosis	5	No	No
23	16 mg	No palpable fibrosis	3	No	No	No palpable fibrosis	3	No	No	Sacrificed at 4 weeks			
24	16 mg	No palpable fibrosis	2	No	No	No palpable fibrosis	3	No	No	No palpable fibrosis	2	No	No
25	160 mg	No palpable fibrosis	2	No	No	2 cm^2^	6	No	No	Sacrificed at 4 weeks			
26	160 mg	No palpable fibrosis	3	No	No	4 cm^2^	7	No	No	4 cm^2^	7	No	No
27	160 mg	No palpable fibrosis	4	No	No	2 cm^2^	5	No	No	Sacrificed at 4 weeks			
28	160 mg	No palpable fibrosis	4	No	No	2 cm^2^	5	No	No	2 cm^2^	5	No	No
29	160 mg	2 cm^2^	4	No	No	3 cm^2^	4	No	No	Sacrificed at 4 weeks			
30	160 mg	2 cm^2^	5	No	No	3 cm^2^	7	No	No	3 cm^2^	7	No	No
31	160 mg	No palpable fibrosis	2	No	No	2 cm^2^	5	No	No	Sacrificed at 4 weeks			
32	160 mg	No palpable fibrosis	3	No	No	4 cm^2^	6	No	No	4 cm^2^	6	No	No
33	160 mg	No palpable fibrosis	2	No	No	4 cm^2^	4	No	No	Sacrificed at 4 weeks			
34	160 mg	No palpable fibrosis	3	No	No	4 cm^2^	7	No	No	4 cm^2^	7	No	No
35	160 mg	No palpable fibrosis	2	No	No	2 cm^2^	7	No	No	Sacrificed at 4 weeks			
36	160 mg	No palpable fibrosis	3	No	No	3 cm^2^	6	No	No	3 cm^2^	7	No	No
37	300 mg	2 cm^2^	4	No	No	4 cm^2^	7	No	No	Sacrificed at 4 weeks			
38	300 mg	No palpable fibrosis	4	No	No	2 cm^2^	8	No	No	3 cm^2^	8	No	No
39	300 mg	No palpable fibrosis	3	No	No	3 cm^2^	6	No	No	Sacrificed at 4 weeks			
40	300 mg	2 cm^2^	3	No	No	4 cm^2^	5	No	No	4 cm^2^	5	No	No
41	300 mg	No palpable fibrosis	3	No	No	2 cm^2^	8	No	No	Sacrificed at 4 weeks			
42	300 mg	No palpable fibrosis	3	No	No	3 cm^2^	8	No	No	3 cm^2^	8	No	No
43	300 mg	No palpable fibrosis	2	No	No	3 cm^2^	7	No	No	Sacrificed at 4 weeks			
44	300 mg	2 cm^2^	5	No	No	4 cm^2^	7	No	No	4 cm^2^	6	No	No
45	300 mg	2 cm^2^	5	No	No	4 cm^2^	8	No	No	Sacrificed at 4 weeks			
46	300 mg	No palpable fibrosis	4	No	No	3 cm^2^	6	No	No	3 cm^2^	6	No	No
47	300 mg	No palpable fibrosis	3	No	No	2 cm^2^	7	No	No	Sacrificed at 4 weeks			
48	300 mg	2 cm^2^	4	No	No	4 cm^2^	7	No	No	4 cm^2^	7	No	No

### Histological and Histomorphometric Analysis

**Figure 2:**
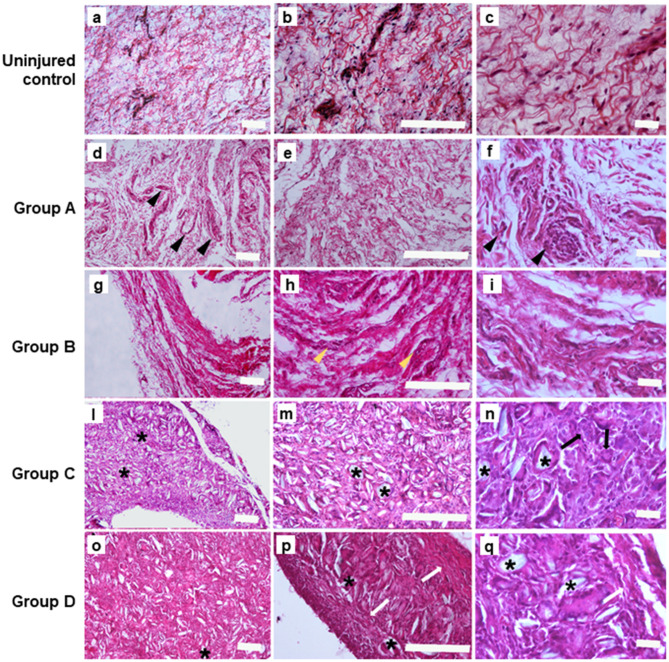
Hematoxylin&Eosin staining performed on the samples at 4 weeks. **(A–C)** Uninjured lesions (Scale bars: a, b 100 μm; c 20 μm); **(D–F)**. Group A: control lesions obtained only by incision and milling, note the presence of leucocytes (pointed arrow) (Scale bars: d, e 100 μm; f 20 μm); **(G–I)**. Group B: lesions obtained by incision an injection of 16 mg/ml of talc (Scale bars: g, h 100 μm; i 20 μm); in **(H)** yellow pointed arrows depict the reactive vascular component; **(L–N)**. Group C: lesions obtained by incision with 160 mg/ml of talc injection; note the presence of talc crystal (*) and multigiant cells (->) (Scale bars: l, m 100 μm; n 20 μm); **(O–Q)**. Group D: Lesions obtained by incision with injection of and 300mg/ml of talc, note the presence the presence of fibrotic capsule (white arrow) around the talc residuals (*) (Scale bars: o, p 100 μm; q 20 μm).

H&E staining and Masson's trichrome staining were conducted to clarify histological differences of adhesion (fibrosis) strength. In the ininjured control H&E stain sample (Figures 2A–C), we observed occurrences of loose connective tissue rich in fibroblasts and with smooth collagen fibers as well as reticular fibers. In the section obtained by incision and scratch (i.e., control) connective tissue was moderately loose but, at higher magnification, the main presence of fibroblasts and leucocytes) was evident (Figures 2D–F). A reactive vascular component and a more fibrous connective tissue with irregular thick collagen, fibers, were well-defined in the lesions obtained by incision and injection of 16 mg/ml of talc (Figures 2G–I); a high level of talc crystal was observed in the lesions obtained by incision and injection of 160 mg/ml, and the presence of multigiant cells was noted at a higher magnification (Figures 2L–N). On the contrary, we noted the presence of fibrotic capsules around the talc with collagen nodules in the lesions obtained by incision and injection of 300 mg/ml of talc and, at higher magnification several macrophages were evident (Figures 2O–Q).

**Figure 3:**
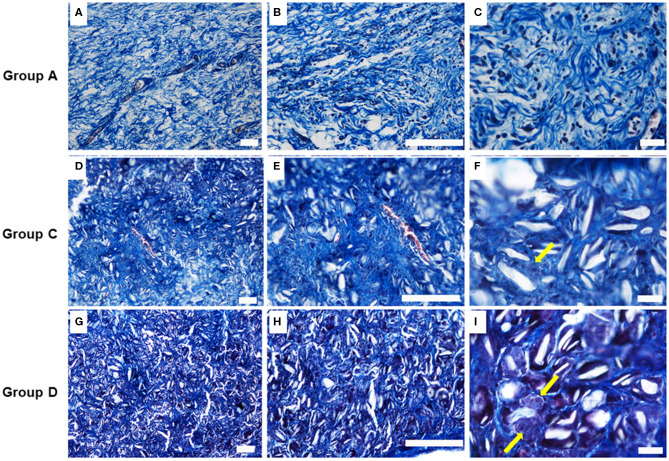
Masson's trichrome stain images on the samples at 4 weeks. **(A–C)** Group A: control lesions (Scale bars: a, b 100–c 20 μm); **(D–F)** Group C: Lesions obtained by incision with injection of 160 mg/ml of talc (Scale bars: d, e 100–f 20 μm); **(G–I)** Group D: Lesions obtained by incision with injection of 300 mg/ml of talc (Scale bars: g, h 100–i 20 m). Note the presence at higher magnification, of multigiant cells (yellow arrows).

Talc crystals and elastic fibers in the adhesion tissues were then stained blue using Masson's trichrome and analyzed by sections from incision and injection of 160 and 300 mg/ml. The presence of multigiant cells was evident together with a leukocyte infiltration mainly represented by macrophages, was also detected (Figure 3). On the contrary, the histological analysis performed on control lesion sections showed the presence of relatively loose connective tissue, rich in fibroblasts and smooth collagen fibers as well as reticular fibers with several vascular structure and leucocytes (Figures 3A–C).

Immunohistochemical staining (data not shown) on collagen type I, III, and IV demonstrated a rise in the collagen type I-III ratio compared to normal murine skin with alphaSMA-positive myofibroblasts. These data indicated talc-induced inflammation and its contribution to the development of adhesion (fibrosis) tissue and irruption of the immune cells.

The ingression of macrophages as well as myofibroblasts and elastic fiber deposits, were observed in the murine model at high concentration of talc. These findings show the rodent model as an example of foreign-body-induced adhesion tissues in humans.

**Figure 4:**
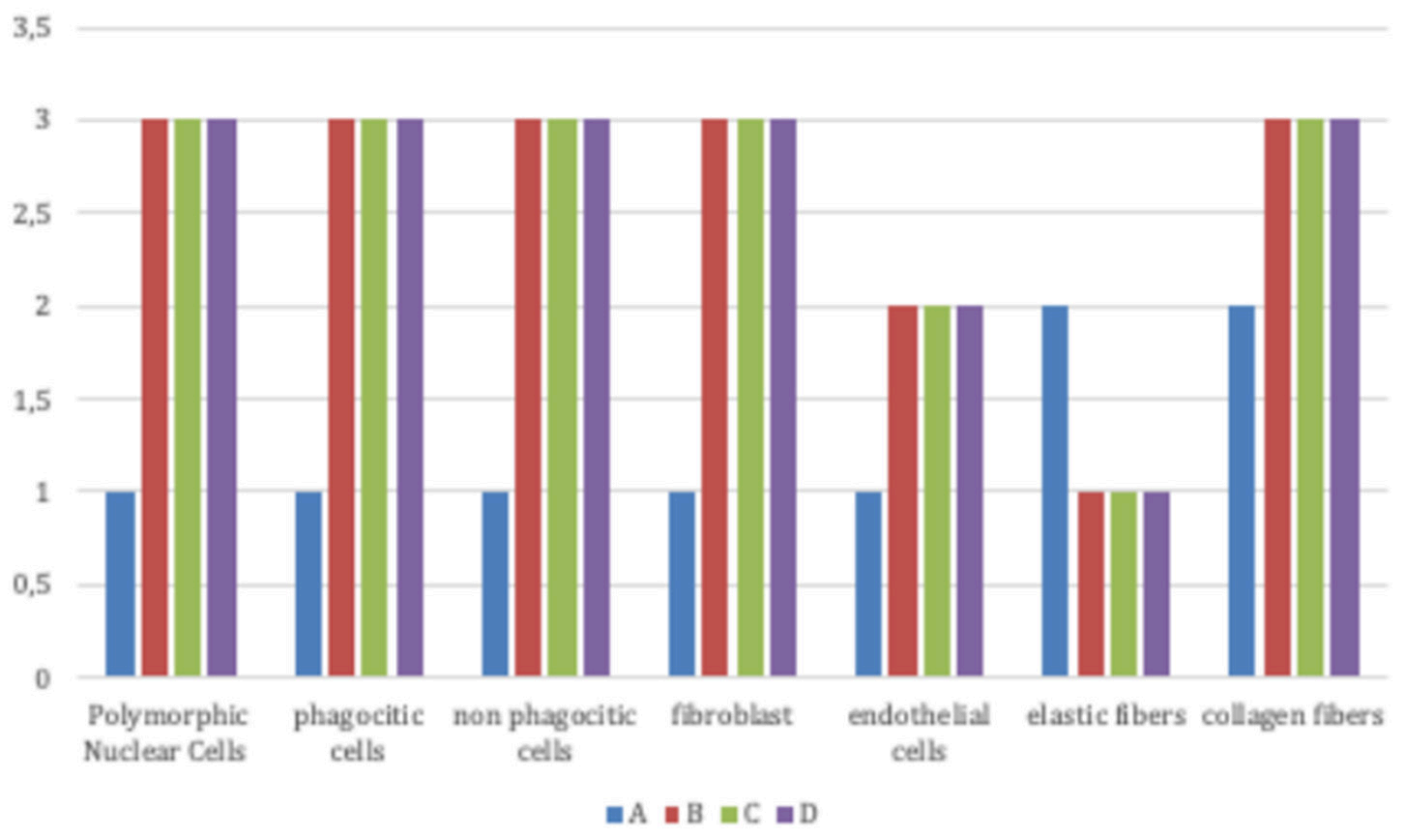
Signs of inflammatory condition in all groups (Group A: control lesions; Group B: 16 mg/ml talc injection; Group C: 160 mg/ml talc injection; Group D: 300 mg/ml talc injection) at 4 weeks: high infiltration of granulocytes, macrophages, polymorphic nuclear cells (PMNs) and non-polymorphic nuclear cells, high fibroblasts and endothelial cells, few elastic fiber, and high collagen fibers were present.

Signs of an inflammatory condition (Figure 4) were detected in all groups after treatment: high infiltration of granulocytes, macrophages, polymorphic nuclear cells (PMNs) and non-polymorphic nuclear cells, high fibroblasts and endothelial cells, few elastic fiber and high collagen fibers were present as well as polymorphic nuclear cells (PMNs) and non-phagocytic cells (lymphocytes).

### Real-Time PCR Array Analysis

We performed real-time PCR array analysis to assess the primary molecules within the wound healing phase.

**Figure 5:**
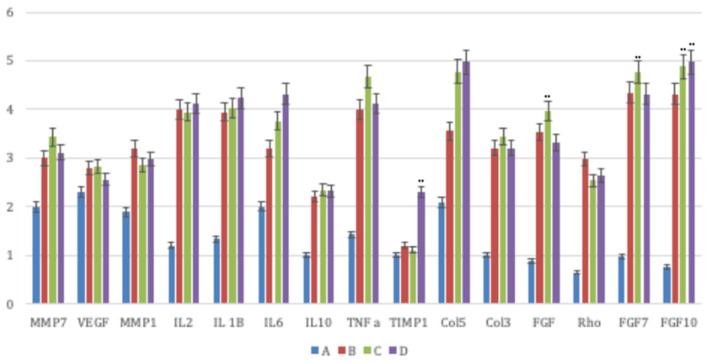
The principal molecules involved in the wound healing process were investigated by means of a real-time PCR array analysis in all groups (Group A: control lesions; Group B: 16 mg/ml talc injection; Group C: 160 mg/ml talc injection; Group D: 300 mg/ml talc injection) at 4 weeks. Regarding the ECM components, the treated samples showed a greater expression of collagens (type 5 and 3 mRNA) compared to control. Concerning the remodeling enzymes, all matrix metalloproteinases (MMPs) displayed a significant increase. Concerning the inflammatory cytokines the treated samples exhibited a greater expression of inflammatory interleukin (IL) 1 and a lesser expression of anti-inflammatory cytokines (IL10). Moreover, they showed high levels of growth factor transcripts, such as the fibroblast growth factor (FGF) 7, FGF10, transforming growth factor (TGF) A, TGFB1, and vascular endothelial growth factor (VEGF). Repeated-measures ANOVA with a *post-hoc* analysis using Bonferroni's multiple comparison. *T* tests were used to determine significant differences (*p* < 0.05). *p < 0.05; **p < 0.01; ***p < 0.001. Repeatability was calculated as the standard deviation of the difference between measurements.

ECM components were specifically analyzed such as cellular adhesion proteins, remodeling enzymes, inflammatory cytokines, and growth factors (Figure 5). As for the ECM components, we observed that the treated samples (B, C, D) a possessed a higher expression of collagens compared to the control samples. The most relevant rise was associated to collagen type 5 and 3 mRNA relative expression. Concerning the remodeling enzymes, we noted a remarkable rise in treatment transcription regarding the matrix metalloproteinases (MMPs).

The analysis of the inflammatory cytokines revealed that the treated samples displayed a more extensive expression in the inflammatory interleukin (IL) 1 and a lower expression in anti-inflammatory cytokines (IL10). We reported, besides, significant levels of growth factor transcripts, such as the fibroblast growth factor (FGF) 7, FGF10, transforming growth factor (TNF) A, and vascular endothelial growth factor (VEGF).

## Discussion

Wound healing in adult mammalians is poorly understood but three overlaying phases are certainly involved—inflammation, proliferation/tissue formation, and remodeling—that results in the formation of a scar (Takeo et al., 2015). Mature scars are largely composed of fibroblasts and type I collagen. This fibrotic “patch” response to injury restores tissue integrity but fails to recapitulate the form and function of the native tissue. Research is currently focusing on the processes of underlying fibrosis to curtail the mechanism and promote regenerative healing (Padmanabhan et al., 2019). Fibrosis in response to injury is not limited to the skin but occurs in almost all adult tissues, specifically idiopathic pulmonary fibrosis (IPF) may be a consequence of myocardial infarction Fibrosis is most apparent in the skin and in dysregulated form will lead to the generation of hypertrophic scars or keloids (Padmanabhan et al., 2019).

Many of the models of hypertrophic scarring consider the pathological features of the hypertrophic scar as opposed to the reproduction process that results in the development of the hypertrophic scarring. Extensive studies have been conducted to define hypertrophic scars and keloids as individual disorders as far as histologic uniqueness and clinical features (Cameron et al., 2014) are concerned.

To the best of our knowledge, consensus is lacking as regards the association of collagen subtypes to hypertrophic scar tissue and the role of key cell types or well-known cytokines as TNF alfa, IL1, IL6, IL10, FGF (Komi et al., 2019). As a consequence, the attention dedicated to the hypertrophic scar phenotype would shift to the fibroproliferative mechanism yielding hypertrophic scarring in an adequate animal model.”

The present study may be the first to describe a rodent model of subcutaneous fibrosis. We considered a simple animal model to assess the novel injection administration, to evaluate besides, the adhesion-promoting compounds as well as defining the temporal sequence and correct dosage that may guarantee an efficient adhesion formation.

Histologically, adhesion tissues (subcutaneous fibrosis) presenting with dense, uniform collagen bundles, and perivascular infiltration as well as vascular wall enlargement are typical histologic features observed in subcutaneous inflammation in humans. Immunohistochemical investigations have previously demonstrated that infiltration at a cellular level will primarily contain inflammatory interleukin. Our model generated histopathologic descriptions regarding human hypertrophic scarring, attributing features such as dermal inspissation owing to collagen deposits, parallel collagen fiber orientation, collagen whorls and inflammatory cell infiltrates. The modifying composition of the extracellular matrix along with collagen fibrillogenesis are important factors that concern fibrotic disease (Karsdal et al., 2013), there is modification in the collagen I to collagen III association within hypertrophic scarring. This could be due to the high concentration of MMPs while in hypertrophic scarring less MMPs are active but TIMPs are expressed at higher levels, this results in accumulation of collagen. However, investigations regarding the modifications are inconsistent (Xue and Jackson, 2015).

Interestingly, our observations suggested the efficient adhesive potential of talc as regards the subcutaneous cavity. Moreover, this potential increased with dose increments. We reported successful adhesion tissue in all animals at 4 weeks subsequent to talc administration. Fibrotic volume was greater in the high-dose groups than in the low-dose groups. However, no significant differences were observed regarding adhesive strength of tissue at 4 and 6 weeks. We recorded greater volume and strength of adhesion tissue at 4 compared to the 2-weeks interval subsequent to injection, but we reported no further change between the 4 and 6 weeks interval. These findings suggest that the 4-weeks time course sufficed to promote the highest rate of adhesions. Furthermore, indications suggest that 300 mg of talc administration for a duration of 4 weeks was sufficient to enhance fibrotic tissue in rats.

At the onset of a cutaneous injury, the accumulation of phagocytic cells as macrophages induce a release of proinflammatory and immunomodulatory mediators that has been evaluated by means of gene expression. Our results confirmed the expression of TNF-α and inflammatory mediators, including VEGF, interleukin (IL)-6, and IL-1, that contribute to increase of endothelial permeability and vasodilation, and facilitate migration of inflammatory cells (mainly monocytes and neutrophils to the site of injury). Moreover, we evaluated the stimulation of fibroblast proliferation during the proliferative phase via IL-10, and basic fibroblast growth factor (bFGF) to produce a new extracellular matrix (ECM) such as collagene type 5 and 3.

During cutaneous wound healing, matrix metalloproteinases remodel the ECM from type III to type I collagen while myofibroblasts mediate wound contraction. During dysregulated fibrosis, cells have been shown to excessively deposit ECM, as in the treated samples herein that showed a greater expression of collagens compared to the control group. We, reported besides, an important rise in collagen type 5 and 3. Driskell et al. demonstrated that reticular fibroblasts in the lower dermis were primarily responsible for fibrosis (Driskell et al., 2013). The present study is in line with the literature (Liu and Zhang, 2008; Corriveau et al., 2009), regarding reports of increased collagen type 3 and type 5 deposition in early fibrosis and increased collagen type I expression in the late stages of the disease. These findings demonstrated that the overproduction of collagen type 5 in particularly severe skin-thickening cases, may justify occurrences of skin fibrosis. Moreover, we may confirm the importance of over-expression in collagen type 5 and type 3 in fibrosis. The increased amounts of collagen type V and type III mRNA expression observed herein indicate transcription regulation most likely attributable to TGF-beta1. Investigations have demonstrated that TGF-beta signalizing is able to increase the expression of collagen in the skin and dermal fibroblast cultures (Sargent et al., 2010). The treatment samples of our study revealed increased levels of growth factors such as FDF7, FGF10, TGF-beta, and VEGF. In particular, VEGF showed a profibrotic effect by multiple mechanisms (Tanaka et al., 2019). The use of talc enhanced the fibroproliferative pathway typical of hypertrophic scarring resulting in scars that directly activated inflammatory and fibrotic mediators fundamental to the human hypertrophic scarring process which is devoid of epidermal injury. In addition, proinflammatory factors, for example interleukin (IL)-1α, IL-1β, IL-6, and tumor necrosis factor-α are overexpressed in keloid, which proposes that the gene expression of these factors in the skin are responsive to trauma. Chronic inflammation, can be favored resulting in invasive keloid growth. Furthermore, the overexpression of proinflammatory factors indicates that keloids and hypertrophic scars are inflammatory conditions of skin, particularly of the reticular dermis (Ogawa, 2017). Based on these considerations, it is obvious that medical doctors cannot regulate the genetic risk factors of a pathological scar. Nevertheless, they can use a number of treatments that, remarkably, work by reducing inflammation like hyaluronic acid (Gao et al., 2019; Riccio et al., 2019), dermal micrograft (Svolacchia et al., 2016; Jimi et al., 2017b). The etiology of skin fibrosis is unknown, albeit investigations proposing unknown antigens as the cause of T-cell activation thus provoking the above-mentioned mechanisms of disease onset. The analysis of inflammatory cytokines in our treated samples revealed a higher expression of inflammatory interleukin (IL) 1 and a lower rate of anti-inflammatory cytokines (IL10). For this reason, Park et al. (2018) demonstrated the contribution of IL-1 in fibrosis by prompting IL-6 and TGF-beta expression in skin fibroblasts yielding a higher fibrotic expression in induced murine models. Moreover, this fibrosis model could help physicians to identify the most useful type of treatment for different clinical conditions of fibrosis such as cellulite and hypertrophic fibrosis that include Ledderhose's syndrome, Peyronie's disease, or more frequently Dupuytren's disease.

## Conclusion

Hypertrophic scarring may be generated via a new model specifically with the use of talc to enhance dermal fibroproliferation. The innovative technique is able to reveal morpho-functional features of human hypertrophic scars to investigate scar formation and assess potential anti-scarring therapies. The talc model may be adopted to better understand the complexities that regard the formation of hypertrophic scars and to foresee preclinical use of anti-scarring treatment.

## Data Availability Statement

All datasets presented in this study are included in the article/supplementary material.

## Ethics Statement

The animal study was reviewed and approved by I.N.R.C.A./I.R.R.C.S. Ethical Committee (No. 1CHPL/08-13).

## Author Contributions

AM contributed conception and design of the study. AM, MM-B, VR, FO, and BZ performed experiments. NZ organized the database. FD performed the statistical analysis. AM and FD wrote the first draft of the manuscript. FD and BZ wrote sections of the manuscript. AM, FD, and MR approved final version of manuscripts. All authors contributed to manuscript revision, read, and approved the submitted version.

## Conflict of Interest

The authors declare that the research was conducted in the absence of any commercial or financial relationships that could be construed as a potential conflict of interest.
